# Ablation of EIF5A2 induces tumor vasculature remodeling and improves tumor response to chemotherapy via regulation of matrix metalloproteinase 2 expression

**DOI:** 10.18632/oncotarget.2236

**Published:** 2014-07-23

**Authors:** Feng-Wei Wang, Mu-Yan Cai, Shi-Juan Mai, Jie-Wei Chen, Hai-Yan Bai, Yan Li, Yi-Ji Liao, Chang-Peng Li, Xiao-Peng Tian, Hsiang-Fu Kung, Xin-Yuan Guan, Dan Xie

**Affiliations:** ^1^ State Key Laboratory of Oncology in South China; Collaborative Innovation Center for Cancer Medicine; Sun Yat-sen University Cancer Center, Guangzhou, China; ^2^ Department of Pathology, Sun Yat-sen University Cancer Center, Guangzhou, China; ^3^ State Key Laboratory of Oncology in South China, the Chinese University of Hong Kong, Hong Kong, China; ^4^ Department of Clinical Oncology, the University of Hong Kong, Hong Kong, China

**Keywords:** Hepatocellular carcinoma, Eukaryotic translation initiation factor 5A2, Matrix Metalloproteinase 2, Vasculature remodeling, Chemotherapy

## Abstract

Hepatocellular carcinoma (HCC) is a highly vascularized tumor with poor clinical outcome. Our previous work has shown that eukaryotic initiation factor 5A2 (EIF5A2) over-expression enhances HCC cell metastasis. In this study, EIF5A2 was identified to be an independent risk factor for poor disease-specific survival among HCC patients. Both *in vitro* and *in vivo* assays indicated that ablation of endogenous EIF5A2 inhibited tumor angiogenesis by reducing matrix metalloproteinase 2 (MMP-2) expression. Given that MMP-2 degrades collagen IV, a main component of the vascular basement membrane (BM), we subsequently investigated the effect of EIF5A2 on tumor vasculature remodeling using complementary approaches, including fluorescent immunostaining, transmission electron microscopy, tumor perfusion assays and tumor hypoxia assays. Taken together, our results indicate that EIF5A2 silencing increases tumor vessel wall continuity, increases blood perfusion and improves tumor oxygenation. Additionally, we found that ablation of EIF5A2 enhanced the chemosensitivity of HCC cells to 5-Fluorouracil (5-FU). Finally, we demonstrated that EIF5A2 might exert these functions by enhancing MMP-2 activity via activation of p38 MAPK and JNK/c-Jun pathways. *Conclusion:* This study highlights an important role of EIF5A2 in HCC tumor vessel remodeling and indicates that EIF5A2 represents a potential therapeutic target in the treatment of HCC.

## INTRODUCTION

Hepatocellular carcinoma (HCC) is one of the most prevalent and lethal cancers worldwide [1]. In the majority of cases, active angiogenesis and a high incidence of intrahepatic metastases result in poor survival. HCC patients diagnosed at an advanced stage, typically respond poorly to traditional therapies as well as to sorafenib, a small molecular inhibitor with efficacy against multiple tyrosine kinases. Consequently, the clinical treatment of HCC remains challenging [2].

In 2001, we isolated a putative oncogene, *eukaryotic initiation factor 5A2 (eIF5A2)*, which is located on chromosome 3q26, and is frequently amplified in multiple human malignancies [3, 4]. Subsequent studies have shown that EIF5A2 may play an important role in malignant transformation, tumor cell proliferation and metastasis [5-8]. Using semi-quantitative RT-PCR and real-time PCR analysis, we demonstrated that EIF5A2 was frequently over-expressed in human HCC tumor tissues compared to matched healthy counterparts. Moreover, compared with matched primary tumor samples, metastatic lesions were found to have higher expression of EIF5A2 by immunohistochemical analysis [6]. Both *in vitro* and *in vivo* studies demonstrate that EIF5A2 plays a pivotal role in promoting HCC cell metastasis by enhancing cell motility and invasion, regulating cytoskeletal organization, and activating the epithelial-mesenchymal transition (EMT) through the Rho/Rac GTPase pathway [6].

Tumor angiogenesis and metastasis are two highly interconnected aspects of tumorigenesis. A recent study from our colleagues showed that EIF5A2 over-expression could promote angiogenesis and metastasis in esophageal squamous cell carcinoma [9]. However, the role of EIF5A2 in HCC angiogenesis remains unclear [10]. In this study, both *in vitro* and *in vivo* approaches show that silencing of endogenous EIF5A2 suppresses angiogenesis induced by HCC cells. EIF5A2 silencing was found to remodel tumor vasculature, increase blood perfusion and sensitize tumor cells to chemotherapeutic agents. In addition, this study sheds light on the molecular mechanisms underlying the effects of EIF5A2 on tumor angiogenesis and vasculature remodeling.

## RESULTS

### Silencing endogenous EIF5A2 inhibits HCC angiogenesis

**Figure 1:**
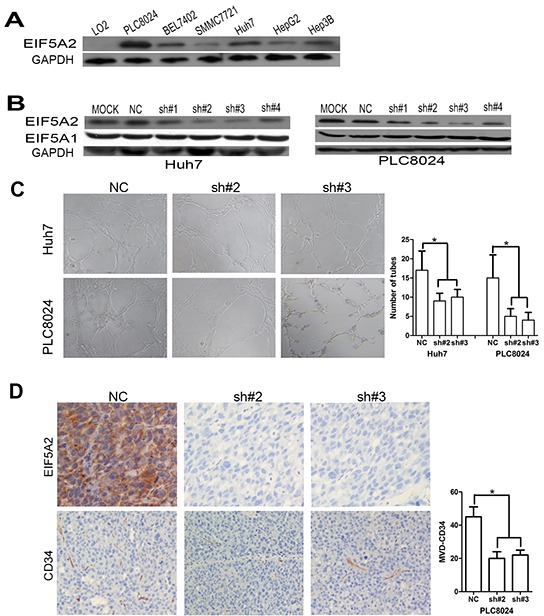
EIF5A2 downregulation represses tumor angiogenesis **(A, B)** Immunoblot analysis of indicated proteins in HCC cell lines. **(C)** EIF5A2 ablation inhibited tube formation of HUVECs induced by HCC cells. Representative images (100x) and quantification of HUVECs cultured on Matrigel-coated plates with 75% conditioned medium from the indicated cell transfected with NC or shRNA-EIF5A2. **(D)** EIF5A2 ablation inhibited tumor angiogenesis in a xenograft model. Sections of tumor tissues derived from PLC8024-NC or PLC8024sh#2 or sh#3 xenografts were stained for EIF5A2 and mouse CD34 by IHC. The three most intensely vascularized areas were evaluated under light microscopy. Representative images (100x) and the quantitative MVD measurement of two groups are presented (^*^, P<0.05).

To determine whether EIF5A2 dysregulation could affect angiogenesis in human HCC, an *in vitro* capillary tube formation assay was employed in PLC8024 and Huh7 HCC cell lines, both of which express high levels of EIF5A2 (Fig. 1A). Endogenous EIF5A2 in Huh7 and PLC8024 cells was silenced through expression of EIF5A2 specific shRNAs. Four independent shRNAs specifically reduced expression of endogenous EIF5A2 in both HCC cell lines (Fig. 1B). Given its comparatively high efficiency of EIF5A2 knockdown, sh#2 and sh#3 was chosen for subsequent studies. Tumor cell-conditioned medium (TCM) from cells transfected with NC, sh#2 or sh#3 was used to supplement the culture medium in which for human umbilical vein endothelial cells (HUVECs) were grown. As shown in Fig.1C, EIF5A2 inhibition suppressed the ability of HCC cells to induce formation of capillary-like structures. In order to further confirm the impact of EIF5A2 on tumor angiogenesis, we injected nude mice subcutaneously with PLC8024 cells stably expressing either sh#2 or sh#3 against EIF5A2 or NC. Compared with the control group, the EIF5A2 knockdown group showed lower micro-vessel density (MVD) as determined by CD34 immunostaining of tumor sample sections (Fig. 1D). Of note, the tumor growth curves of both groups showed that ablation of endogenous EIF5A2 did not significantly impact tumor cell growth, suggesting that the inhibitory function of EIF5A2 silencing on angiogenesis may not be resulted from tumor growth inhibition (Supplementary Fig. 1).

### MMP2 mediates the effect of EIF5A2 on angiogenesis

**Figure 2:**
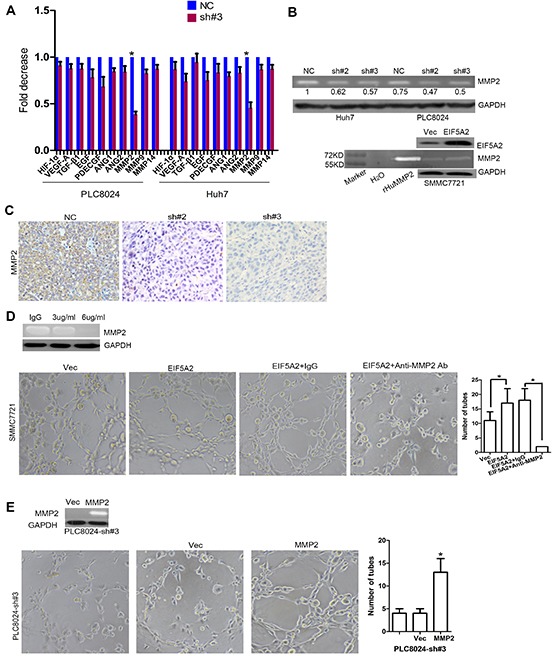
MMP-2 is required for EIF5A2-induced angiogenesis in HCC **(A)** Changes in mRNA expression of the indicated genes in two EIF5A2-suppressed HCC cell lines were assessed by real-time qPCR. **(B)** Analysis of MMP-2 activity in TCM by gelatin zymography. TCM was collected from tumor cells transfected with NC or shRNA-EIF5A2 or with control vector or eIF5A2 over-expression vector. Recombinant human MMP2 (rHuMMP2) as the positive control. **(C)** Analysis of MMP-2 expression in xenograft tumor tissue sections. **(D)** Inhibition of MMP-2 (final concentration of anti-MMP2 antibody: 6ug/ml) repressed the pro-angiogenic effect of EIF5A2 over-expression in SMMC7721 cells. (E) Re-expression of MMP-2 in EIF5A2 knockdown PLC8024 cells restored the pro-angiogenic effect of EIF5A2.

We next explored the underlying molecular mechanisms responsible for the effects of EIF5A2 on angiogenesis. Based on our previous study as well as previously published reports, we used real-time RT-PCR to screen the molecular markers closely related with angiogenesis in HCC [10]. As shown in Fig. 2A, MMP-2 was significantly downregulated at the transcript level in both PLC8024-sh#3 and Huh7 sh#3 cell lines, while other angiogenesis markers exhibited only slight changes in response to EIF5A2 suppression. Furthermore, gelatin zymography demonstrated that, compared with TCM obtained from control cells, TCM from PLC8024-sh#2 or sh#3 and Huh7-sh#2 or sh#3 exhibited a significant reduction in MMP-2 activity (Fig. 2B). Consistent with these results, ectopic EIF5A2 expression in SMMC7721 cells enhanced MMP-2 activity (Fig. 2B). Furthermore, compared with PLC8024-NC group, xenografts tumors of PLC8024-sh#2 or sh#3 also showed decreased MMP-2 expression analyzed by IHC (Fig. 2C). To investigate the requirement for MMP2 in angiogenesis induced by EIF5A2, we employed an MMP-2 blocking assay. As expected, treatment of SMMC7721-EIF5A2 cells with MMP-2 neutralizing antibody (final concentration: 6μg/ml) attenuated the pro-angiogenic effect of EIF5A2 (Fig. 2D). Additionally, MMP2 over-expression in PLC8024-sh#3 cells rescued the effect of EIF5A2 on angiogenesis (Fig. 2E). These data indicate that MMP2 is necessary for EIF5A2-induced angiogenesis.

**Figure 3:**
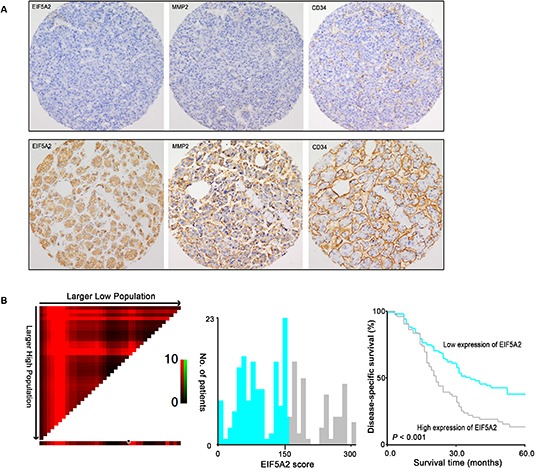
Association among expression of EIF5A2, MMP-2, MVD and its prognostic significance in HCC patients **(A)** Representative images of IHC staining for EIF5A2, MMP-2 and CD34 in two HCC cases (100x). EIF5A2 expression positively correlated with MMP-2 expression and tumor MVD. **(B)** X-tile analysis was employed to determine the cut-point for EIF5A2 expression in HCC. The cut-point (H score=150) highlighted by the black/white circle in the horizontal axis (left panel) was demonstrated on a histogram of the cohort (middle panel), and a Kaplan-Meier plot (right panel).

Immunoreactivity for EIF5A2 was examined primarily in the cytoplasm of tumor cells. The nuclear expression of EIF5A2 was also observed in 36 (36/212) HCC cases. The association between EIF5A2 expression and either MMP2 expression or angiogenesis in 212 HCC patients were further assessed using immunohistochemistry on a tissue micro-array (TMA) platform, which demonstrated that EIF5A2 expression was significantly associated with MMP-2 expression as well as MVD (P=0.022 and P=0.030) (Table. 1 and Fig. 3A). Based on the X-tile analysis, the cut-point for high expression of EIF5A2 was defined when the H scores were above 150. Further correlation analysis showed that EIF5A2 over-expression in HCCs was significantly associated with tumor multiplicity and stage (P<0.05, Table 1). Kaplan–Meier analysis established that patients with high EIF5A2 expression had poorer disease-specific survival compared to those with low EIF5A2 expression (Log-rank test, P<0.001) (Table 2 and Fig. 3B). Multivariate Cox regression analysis identified EIF5A2 expression as an independent risk factor for poor disease-specific survival of HCC patients, with a hazard ratio (HR) of 1.522 (95% confidence interval [CI]: 1.065-2.175) (P=0.021) (Table 3). Moreover, transwell assay indicated that MMP2 was also involved in the inducible effect of EIF5A2 on tumor invasion (Supplementary Fig. 2). Taken together, these findings suggest that EIF5A2 overexpression may promote angiogenesis and invasion of HCC by enhancing MMP2 activity.

**Table 1:** Correlation of IEF5A2 expression with patients' clinicopathological features in primary hepatocellular carcinomas

Variable	EIF5A2 protein			
	All cases	Low expression	High expression	*P* value^1^
Age (years)				0.266
≤ 47.9^2^	105	65 (61.9%)	40 (38.1%)	
> 47.9	107	74 (69.2%)	33 (30.8%)	
Sex				0.432
Female	38	27 (71.1%)	11 (28.9%)	
Male	174	112 (64.4%)	62 (35.6%)	
HbsAg				0.611
Negative	48	30 (62.5%)	18 (37.5%)	
Positive	164	109 (66.5%)	55 (33.5%)	
AFP (ng/ml)				0.773
≤20	67	43 (64.2%)	24 (35.8%)	
>20	145	96 (66.2%)	49 (33.8%)	
Liver cirrhosis				0.665
No	80	51 (63.8%)	29 (36.3%)	
Yes	132	88 (66.7%)	44 (33.3%)	
Tumor size (cm)				0.042
≤5	59	45 (76.3%)	14 (23.7%)	
>5	153	94 (61.4%)	59 (38.6%)	
Tumor multiplicity				0.017
Single	128	92 (71.9%)	36 (28.1%)	
Multiple	84	47 (56.0%)	37 (44.0%)	
Differentiation				0.285
Well-moderate	153	97 (63.4%)	56 (36.6%)	
Poor-undifferentiated	59	42 (71.2%)	17 (28.8%)	
Stage				0.032
I-II	88	66 (73.9%)	23 (26.1%)	
III- IV	124	74 (59.7%)	50 (40.3%)	
Vascular invasion				0.164
Absent	104	73 (70.2%)	31 (29.8%)	
Present	108	66 (61.1%)	42 (38.9%)	
Relapse				0.362
Absent	107	67 (62.6%)	40 (37.4%)	
Present	105	72 (68.6%)	33 (31.4%)	
MMP2				0.022
Low expression	55	43 (78.2%)	12 (21.8%)	
High expression	157	96 (61.1%)	61 (38.9%)	
MVD				0.030
≤79	126	90 (71.4%)	36 (28.6%)	
>79	86	49 (57.0%)	37 (43.0%)	

1 Chi-square test;

2 Mean age.

**Table 2:** Univariate analysis of EIF5A2 expression and clinicopathologic variables in 212 patients with primary hepatocellular carcinoma (log-rank test)

Variables	All cases	RR (95% CI)	P value
Age (years)			0.627
≤ 47.9	105	1.0	
> 47.9	107	1.090 (0.770-1.542)	
Sex			0.045
Female	38	1.0	
Male	174	1.650 (1.012-2.688)	
HbsAg			0.810
Negative	48	1.0	
Positive	164	1.053 (0.692-1.602)	
AFP (ng/ml)			0.000
≤20	67	1.0	
>20	145	2.262 (1.490-3.434)	
Liver cirrhosis			0.891
No	80	0.975 (0.682-1.395)	
Yes	132	1.0	
Tumor size (cm)			0.000
≤5	59	1.0	
>5	153	6.295 (3.688-10.747)	
Tumor multiplicity			0.000
Single	128	1.0	
Multiple	84	3.480 (2.429-4.987)	
Differentiation			0.021
Well-moderate	153	1.0	
Poor-undifferentiated	59	1.534 (1.068-2.203)	
Stage			0.000
I-II	88	1.0	
III- IV	124	5.393 (3.525-8.252)	
Vascular invasion			0.000
Absent	104	1.0	
Present	108	4.923 (3.310-7.322)	
Relapse			0.000
Absent	107	1.0	
Present	105	1.907 (1.330-2.734)	
EIF5A2 expression			0.000
Low	139	1.0	
High	73	1.914 (1.350-2.714)	

CI, confidence interval; HbsAg, hepatitis B surface antigen; AFP, alpha-fetoprotein.

**Table 3:** Cox multivariate analyses of prognostic factors on disease-specific survival

Variables	Hazards ratio	95% CI	*P* value
AFP, ng/ml (≤20*v* >20)	1.341	0.876-2.054	0.177
Tumor size, cm (≤5*v* >5)	2.138	1.158-3.945	0.015
Tumor multiplicity (single *v* multiple)	2.005	1.381-2.911	0.000
Differentiation(well-moderate *v* poor-undifferentiated)	1.453	0.995-2.123	0.053
Stage (I-II *v* III-IV)	2.391	1.455-3.930	0.001
Vascular invasion (absent *v* present)	2.199	1.427-3.388	0.000
Relapse (absent *v* present)	1.318	0.902-1.926	0.154
EIF5A2 expression (low *v* high)	1.522	1.065-2.175	0.021

CI, confidence interval; AFP, alpha-fetoprotein.

### Silencing endogenous EIF5A2 promotes tumor vascular normalization

**Figure 4:**
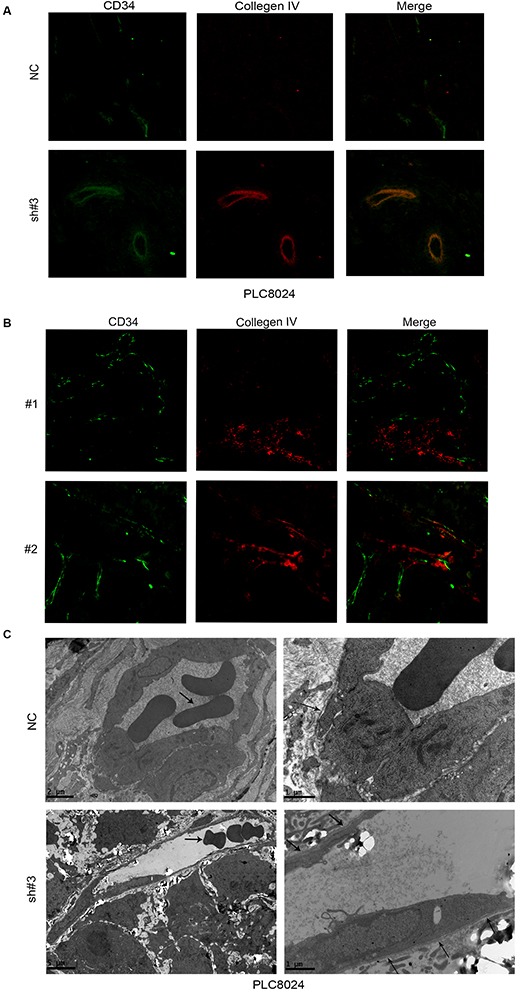
EIF5A2 ablation increases micro-vessel wall continuity and promotes normal endothelial cell lining and differentiation **(A)** Xenograft tumor sections stained for CD34 (green) and collagen IV (red) revealed more continuous BMs in EIF5A2- ablation cells compared to control cells. **(B)** Representative images of human HCC tissue sections stained for CD34 and collagen IV by IF (400x). Twenty cases (10 with high EIF5A2 and MMP-2 expression and 10 with low EIF5A2 and MMP-2 expression), were analyzed for tumor vessel wall continuity by IF. Increased continuity of vessel walls was seen in all cases with low EIF5A2 and MMP-2 expression. For A and B, the three most intensely vascularized areas were evaluated under immunofluorescence microscopy. **(C)** Transmission electron microscopy of vessels in tumors derived from PLC8024 cells transfected with NC or sh#3. Left column, arrowheads indicate red blood cell. Compared with NC group, endothelial cells in the EIF5A2 knockdown group were lined with more continuous BMs (right column, arrowhead). For the NC group, left column, original magnification 5800x; right column, 13500x. For sh#3 group, left column, original magnification 2400x; right column, 13500x.

Blood vessels are comprised of basement membranes (BMs), pericytes and vascular endothelial cells. Vascular BM components are required for the initiation and resolution of angiogenesis [11]. Since type IV collagen, a substrate for MMP-2 and the main component of the vascular BM, is crucial for BM stability and assembly [12, 13]. We hypothesized that EIF5A2 may also affect tumor blood vessel wall remodeling or vessel structure via regulation of MMP-2 activity. Collagen IV and CD34 expression were examined by immunofluorescent staining of xenograft tissue sections. Compared with the control group, tumor micro-vessels in xenografts from EIF5A2 knockdown tumor cells exhibit more continuous and smooth wall, more regular endothelial lining, and morphology similar to normal vasculature (Fig. 4A). Similarly, vessel walls in EIF5A2 and MMP2 low expression human HCC tissue sections were more continuous (Fig. 4B, Supplementary Fig. 3). Transmission electron microscopy further revealed that the vessels in the EIF5A2 knockdown group were more likely to have continuous BMs, well differentiated endothelial cells, and same polarity in their endothelial linings (Fig. 4C). These data suggest that EIF5A2 ablation could induce formation of integrated, continuous vascular walls and normalized endothelial cells lining. Moreover, transmission electron microscopy indicated that the micro-vessels in EIF5A2 knockdown group displayed larger lumens and thinner walls which may enhance blood perfusion and facilitate nutrient and oxygen exchange.

### Downregulation of EIF5A2 increases tumor perfusion and response to chemotherapy

**Figure 5:**
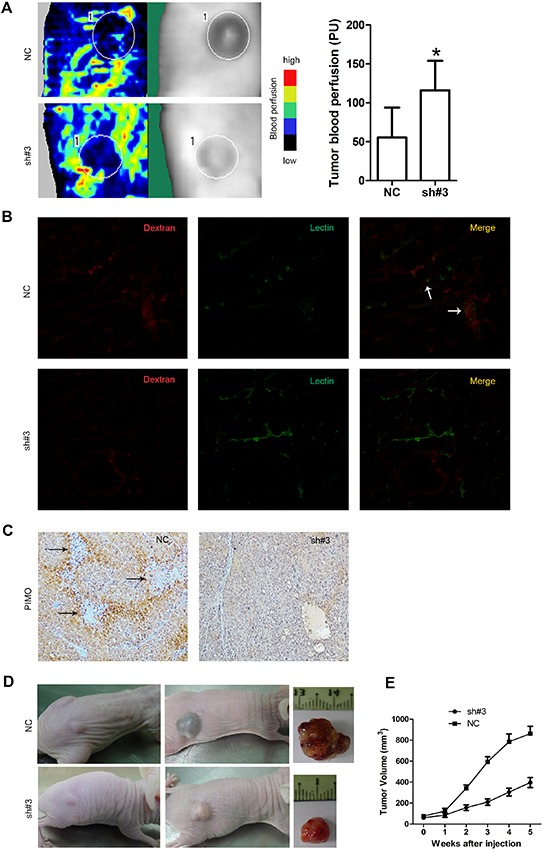
EIF5A2 ablation increases tumor blood perfusion, reduces tumor hypoxia and enhances sensitivity to chemotherapy **(A)** Representative images of Doppler laser imaging of intratumoral blood flow. Left side: Doppler imaging; Right side: the corresponding position of the Doppler imaging on the mice. The mean value of the blood flow is the mean number of the blood flow of six tumors in one group mice. Bar graphs show quantification of corresponding perfusions. P=0.011. **(B)** Increased tumor vessel perfusion and reduced vessel leakiness in the PLC8024-sh#3 group following injection with Texas Red-conjugated Dextran (red) and FITC-conjugated Lectin (green). White arrowheads on merged images indicated sites of leakage. **(C)** Reduced staining for pimonidazole in tumors derived from PLC8024 cells transfected with NC or sh#3. Arrowheads indicated necrosis regions. Cells peripheral to necrotic areas were stained most intensely, indicating severe hypoxia. **(D)** EIF5A2 silencing sensitizes xenografts to 5-fluorouracil. Representative images of xenograft tumors induced by PLC8024-NC or PLC8024-sh#3 in nude mice. Left column, before treatment; middle column, 5 weeks after treatment with 5-FU (intraperitoneal injection, 40mg/kg body weight, once weekly for 5 weeks); right column, representative tumors excised from mice after 5 weeks of treatment. E. Tumor growth curves (N=6 mice per group); tumor volumes were measured on a weekly basis.

We further assessed the effect of EIF5A2 ablation on blood perfusion and the tumor microenvironment. Tumor blood vessel flow, as measured by laser Doppler imaging, was significantly increased in the EIF5A2-silenced group compared to the NC control group (Fig.5A). To further examine intratumoral blood perfusion and leakage, a mouse model was created by injecting FITC-Lectin and Dextran into the tail vein of tumor bearing nude mice. Fluorescent immunostaining of tumor sections indicated that EIF5A2 downregulation increased tumor perfusion while reducing blood leakage (Fig. 5B). Since supplying oxygen is an ancestral function of vessels, we hypothesized that EIF5A2 inhibition would improve tumor oxygenation. IHC staining of the hypoxia-marker pimonidazole revealed a reduction in hypoxia in PLC8024-sh#3 cells (Fig. 5C). We then investigated whether endogenous EIF5A2 ablation could increase the sensitivity of HCC cells to chemotherapy. Twelve nude mice were injected subcutaneously with either PLC8024-NC (n=6) or PLC8024-sh#3 (n=6) cells. When the xenograft tumors reached ~5 mm^3^ in size, mice were injected intraperitoneally with 5-FU (40 mg/kg body weight once per week for 5 weeks). In comparison with the PLC8024-NC group, tumor growth rates and tumor volumes were significantly reduced in the PLC8024-sh#3 group (p<0.05, Fig. 5D and 5E). These data suggested that silencing of endogenous EIF5A2 expression could reduce tumor hypoxia, thus increase the chemosensitivity to 5-FU by remodeling tumor vessels.

### EIF5A2 regulates MMP2 by activating p38 MAPK and JNK/c-Jun pathway

**Figure 6:**
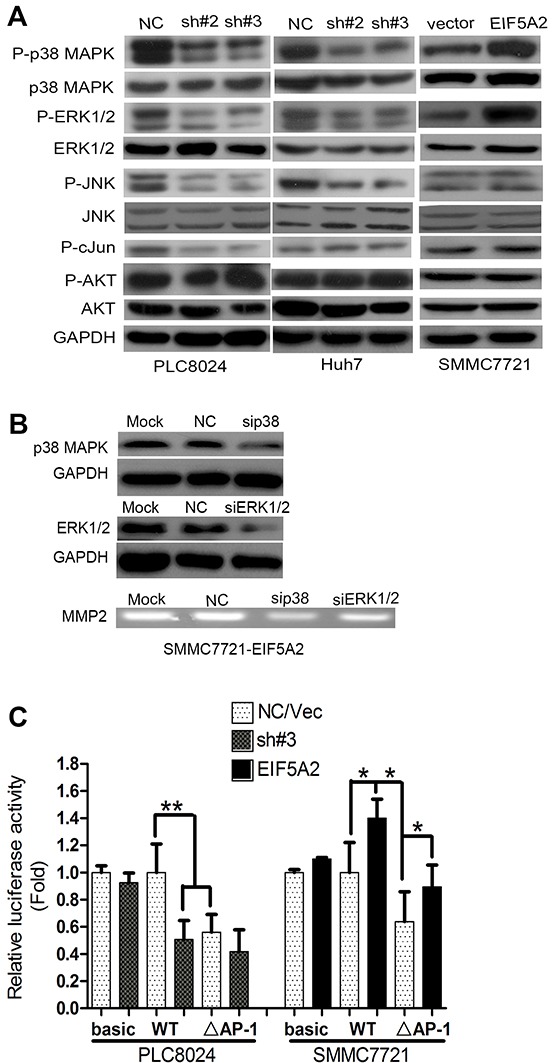
EIF5A2 induces MMP-2 transcription by activating p38 MAPK and JNK/c-Jun pathways **(A)** Immunoblot for indicated mediators of MAPK pathway signaling in cells transfected with NC or sh#3 or control vector or EIF5A2 over-expression vector. **(B)** MMP-2 activity in TCM examined by gelatin zymography. TCM was collected from cells transfected with NC or si-RNA targeting p38 MAPK or ERK1/2. **(C)** Analysis of luciferase activity using MMP-2 promoter luciferase reporter vector in indicated cells. Basic: pGL3-basic; WT: wild type promoter; ΔAP-1: deletion of AP-1 binding site (**, P<0.01; ^*^, P<0.05, T test).

To further explore the mechanisms by which EIF5A2 regulates MMP-2 activity, we evaluated the effects of EIF5A2 knockdown or over-expression on the activation of signal transduction pathways. The MAPK signaling pathway regulates MMP2 expression depending on cellular context, and is mediated by p38 MAPK, ERK1/2, and JNK/c-Jun [14-17]. In agreement with previous findings, our study showed that the levels of phosphorylated ERK1/2 and p38 MAPK were dramatically decreased in both EIF5A2-silenced PLC8024 and Huh7 cells, but increased in EIF5A2-overexpressing SMMC7721 cells (Fig. 6A). Interestingly, phosphorylated JNK and c-Jun were also significantly decreased in EIF5A2-silenced PLC8024 cells, but only slight changes were observed in EIF5A2-silenced Huh7 and SMMC7721 cells ectopically expressing EIF5A2 (Fig. 6A). However, phosphorylated Akt levels remained unchanged in regardless of EIF5A2 expression. To further explore the role of p38 MAPK and ERK1/2 in the induction of MMP-2 activity by EIF5A2, siRNAs targeting to p38 MAPK and ERK1/2 were transfected into the EIF5A2-expressing SMMC7721 cells. MMP-2 activity in the EIF5A2-transfected SMMC7721 cells was shown to be decreased by siRNAs against p38 MAPK but not ERK1/2 (Fig. 6B). To examine whether the c-Jun participates in the induction of MMP-2 activity by EIF5A2, luciferase reporter vector containing the wild-type MMP-2 promoter and a deletion mutation of AP-1 binding site within the MMP-2 promoter (ΔAP-1-MMP-2) were transiently co-transfected with pRL-TK into PLC8024-NC and sh#3 cells or co-transfected with vector or EIF5A2 over-expression vector into SMMC7721 cells [18]. In contrast to the wild-type promoter reporter, the luciferase activity of ΔAP-1-MMP-2 was unchanged between PLC8024-NC and sh#3 cells (Fig. 6C). However, in SMMC7721 cells, EIF5A2 over-expression enhanced luciferase activity driven by the deletion mutant promoter (Fig. 6C). Moreover, the deletion of the AP-1 binding site significantly reduced reporter activity in both cell lines, which indicates that AP-1 is an important regulatory factor for MMP-2 activity (Fig. 6C). Taken together, these results indicated that the stimulation of MMP-2 by the EIF5A2 signaling pathway depended on specific cellular contexts. To further explore whether EIF5A2 regulates MMP-2 expression primarily at the transcriptional level, the MMP-2 3′-UTR (untranslated region) was cloned into the pMIR-REPORT luciferase reporter vector. Dual-luciferase reporter analysis showed that EIF5A2 silencing did not alter the activity of the MMP2 3′-UTR luciferase reporter, indicating that EIF5A2 may regulate MMP-2 gene expression by enhancing binding of transcription factors binding such as AP-1 at the MMP-2 promoter (Supplementary Fig. 4). However, the roles of other potential transcriptional factors in the induction of MMP2 by EIF5A2 still warrant further evaluation.

## DISCUSSION

HCC is a highly vascularized tumor that frequently undergoes intrahepatic metastasis [10]. Despite the development of multimodality treatment approaches, including surgical resection, chemotherapy, radiotherapy, as well as the molecular targeted therapies, the outcome of HCC patients remains poor. Therefore, it is urgent to further define the key molecular mechanisms driving HCC progression and identify new targets for the treatment of HCC. In this study, we show for the first time that the ablation of endogenous EIF5A2 can improve tumor oxygenation and sensitizes to chemotherapy by remodeling tumor vasculature. We also demonstrated that EIF5A2 may exert these functions by enhancing MMP-2 activity via activation of the MAPK pathways.

Since angiogenesis is essential for tumor growth, development of antiangiogenic therapies to suppress tumor progression is an area of intense research focus [19, 20]. In this study, the influences of EIF5A2 on angiogenesis, vascular structure, vessel function and tumor microenvironment in HCC were evaluated using both *in vitro* and *in vivo* approaches. Immunohistochemical staining of CD34 showed that silencing endogenous EIF5A2 could reduce tumor MVD, a marker for angiogenesis. Consistent with the role of EIF5A2 in melanoma [21], functional studies as well as the clinical data indicate that EIF5A2 over-expression in HCC can promote tumor angiogenesis and tumor cell invasion. Accumulating evidences show that MMPs play an important role in tumor invasion, metastasis and angiogenesis. However, MMP2, MMP9 and MMP14 [22] are the three major MMPs reported to be involved in tumor angiogenesis. Therefore, we tested the RNA levels of MMP2, MMP9 and MMP14 in EIF5A2 ablation cell lines to explore whether they take part in the regulation of HCC angiogenesis by EIF5A2. Previous studies also reported that MMP14 promoter's transcriptional activity could be regulated by SP1, NFAT1c and Egr-19 [23, 24], while the expression of MMP2 and MMP9 were mediated by cytokines via activation of transcription factors (TFs) such as AP-1, which can be regulated by MAPK signaling. However, MMP-2 was found to be an essential molecule in determining endothelial cell (EC) fate, paradoxical effects on both angiogenesis and cell death. Two major apoptotic pathways in ECs, caspases and p38 MAPK, enhance MMP-2 synthesis and affect EC behavior via different activation form of MMP-2 [25, 26]. Our data did show that MMP2 but not MMP9 and MMP14 level was significantly changed in EIF5A2 ablation cells. Taken together, these evidence may at least partly support that MMP2 is selectively involved in EIF5A2-related mechanism, but no for MMP9 and MMP14. Nonetheless, the underlying mechanism by which EIF5A2 regulates MMPs requires further investigation.

Collagen IV, the main component of the vascular BMs and the substrate of MMP-2, is important for BM integrity and structural organization. BMs are essential for proper endothelium organization, migration and proliferation [27]. Therefore, we examined the effect of EIF5A2 silencing on tumor vessel wall remodeling, vascular function and tumor microenvironment. CD34 and collagen IV co-staining of xenograft tissue sections and human HCC tissue sections demonstrated that tumor vessel walls in the EIF5A2 low expression group were more smooth and continuous. Transmission electron microscopy further verified that EIF5A2 ablation induced well-differentiated phenotype in endothelial cells. BMs function provides a supporting substratum for epithelial sheets and maintain cell polarity in epithelia [27]. Consistent with these findings, our results indicated that continuous vascular BMs might also provide a supporting scaffold for endothelial cell lining and differentiation, which in turn makes vascular walls thinner and facilitates material exchange of oxygen and nutrients. Furthermore, we found that EIF5A2 silencing could increase blood perfusion, reduce tumor hypoxia and thus improve the sensitivity to chemotherapy.

Despite significant inhibition of tumor angiogenesis by EIF5A2 ablation, obvious suppression of liver tumor growth was not observed in this study, indicating that agent targeting angiogenesis itself may not be able to sufficiently inhibit tumor growth. Consistent with our findings, recent clinical data showed that traditional antiangiogenic agents may have limited efficacy in the treatment of cancer, with only minimal improvements in overall patient's survival [28]. To investigate the discrepancies between initial predictions and the clinical data, Conley and colleagues demonstrated that the antiangiogenic agents might enrich the population of breast cancer stem cells by generating intratumoral hypoxia [29]. Studies by Mazzone *et al.* using transgenic mouse model demonstrated that endothelial lining normalization and vessel maturation could improve tumor perfusion and oxygenation, inhibiting tumor cell metastasis and thus improving tumor response to chemotherapy [30, 31]. These findings suggest that potential effects on tumor vasculature structure, function and tumor microenvironment should be considered in the design and development of new antiangiogenic agents. Taken together with the findings presents here, these data strongly suggest EIF5A2 is a promising molecular target for anti-HCC therapy.

We further investigated the underlying mechanisms by which EIF5A2 regulated MMP-2 activity. Luciferase reporter assays and siRNA specifically targeting MAPK pathway mediators showed that EIF5A2 could promote MMP-2 activity by activating the JNK/c-Jun and p38 MAPK pathway. We also found that EIF5A2 ablation or over-expression had no significant effect on the expression of TIMP2, the natural inhibitor of MMP2 (Data not shown). Fang *et al.* found that miR-29b could suppress tumor angiogenesis and metastasis by directly targeting MMP-2 [32]. Luciferase reporter gene assays showed that EIF5A2 regulates MMP-2 primarily at the transcriptional level through its promoter activity but not its 3′UTR, indicating that microRNAs do not play an essential role in the interplay between EIF5A2 and MMP2 in HCC.

Tumor blood vessels are characterized by distorted vessel shape, capillary sprouting, lack of proper pericyte coverage and leakiness, which facilitate extravagation of tumor cells [19]. Our previous study showed that EIF5A2 could promote HCC cell metastasis by inducing tumor cell EMT. The data presented in this study suggested that EIF5A2 deregulation may play a vital role in tumor angiogenesis, remodeling of vascular structure and chemotherapy sensitivity of HCC. Recent other studies also showed that EIF5A2 play an important role in chemoresistance in breast cancer and promote bladder cancer cell aggressiveness [33-35]. Taken together, ablation of EIF5A2 may represent a promising anti-cancer strategy, targeting the crossroads of angiogenesis and metastasis in HCC.

## MATERIALS AND METHODS

### Patients and tissue microarray (TMA)

Formalin-fixed, paraffin-embedded tissues of 212 HCC patients, who underwent initial surgical resection between March 2003 and August 2006, were obtained from the archives of the Department of Pathology of the First Affiliated Hospital, Sun Yat-sen University (Guangzhou, China). Clinicopathological characteristics for the HCC cohort were detailed in Table 1. Tumor differentiation was determined based on the criteria proposed by Edmonson and Steiner [36]. Tumor stage was defined according to the tumor-node-metastasis (TNM) classification system from the American Joint Committee on Cancer/International Union Against Cancer [37]. The Institute Research Medical Ethics Committee of Sun Yat-sen University granted approval for this study.

TMAs were constructed in accordance with a previously-described method [38].

### Cell lines

Six HCC cell lines (SMMC-7721, Hep3B, PLC8024, Huh7 BEL7402 and HepG2) were selected and cultured in this study. All HCC cell lines and immortalized human liver cells LO2 were cultured in high-glucose DMEM (Gibco BRL, Grand Island, NY) supplemented with 10% fetal bovine serum. Human umbilical vein endothelial cells (HUVECs) was a generous gift from Dr Li-bing Song (Sun Yat-sen University Cancer Center, Guangzhou, China) [39]. HUVECs were cultured as previously described [34]. Primary HUVECs were used at passages 2-7 in all experiments.

### Quantitative real-time RT-PCR (qRT-PCR) and western blot

Real-time RT-PCR was performed using an ABI PRISM 7500 Sequence Detection System (Applied Biosystems) with 2x SYBR green master mix (Applied Biosystems, Foster City, CA) using the 2-Δ ΔCT method. qRT-PCR primers are listed in Table 4. Western blotting was performed according to standard protocols with antibodies for AKT (cat.2920, Cell Signaling Technology, Beverly, MA), phospho-ser473-AKT (cat.4060, CST), ERK1/2 (cat.4696, CST), phosphor-T202/ Y204-ERK1/2 (cat.4370, CST), p38 MAPK (cat.9212, CST), phospho-T180/Y182-p38 MAPK (cat.4511, CST), SAPK/JNK (cat.9268, CST) and phospho-T183/Y185-SAPK/JNK (cat.4668, CST), MMP-2 (cat.10373-2-AP, Proteintech Group, Chicago, USA), phospho-S63-cJun (cat. ab32385, Abcam, USA) and GAPDH (cat. BM1985, Boster, Wuhan, China).

**Table 4:** Primer sequences used for qRT-PCR

Name	Sequence
ANG1-F	5′-ATGGACTGGGAAGGGAACCGAGC-3′
ANG1-R	5′-GGGGCCACAAGCATCAAACCACC-3′
ANG2-F	5′-TAAGCAGCATCAGCCAACCAGGAAA-3′
ANG2-R	5′-TTTGTGTTCTGCCTCTGTGGATAGTA-3′
VEGFA-F	5′-CCTGTGTGCCCCTGATGCGATG-3′
VEGFA-R	5′-GCTTTCGTTTTTGCCCCTTTCCCT-3′
HIF1A-F	5′-GCACAGAAGCAAAGAACCCATTTTC-3′
HIF1A-R	5′-GGCAGTGGTAGTGGTGGCATTAG-3′
EGF-F	5′-TGCCAGCTGCACAAATACAGAGGG-3′
EGF-R	5′-CATCGTGGGACAGGGGACATTCA-3′
PD-ECGF-F	5′-TCCTGCGGACGGAATCC-3′
PD-ECGF-R	5′-TGAGAATGGAGGCTGTGATGAG-3′
MMP2-F	5′-CTTCCAAGTCTGGAGCGATGT-3′
MMP2-R	5′-TACCGTCAAAGGGGTATCCAT-3′
MMP9-F	5′-GGGACGCAGACATCGTCATC-3′
MMP9-R	5′-TCGTCATCGTCGAAATGGGC-3′
MMP14-F	5′- GGAATAACCAAGTGATGGATGG-3′
MMP14-R	5′- TTGTTTCCACGGAAGAAGTAGG-3′
GAPDH-F	5′-CATGAGAAGTATGACAACAGCCT-3′
GAPDH-R	5′-AGTCCTTCCACGATACCAAAGT-3′

### RNA oligonucleotides and vectors

Small interfering RNA (siRNA) duplexes specific for p38 MAPK and MMP2 were purchased from Genepharma (Shanghai, China), and siRNA specific for ERK1/2 were purchased from Cell Signaling (Beverly, MA). The negative control RNA duplex (NC) for siRNA was not homologous to any human genome sequence.

Vectors: EIF5A2 specific shRNA vectors were purchased from Sigma. pcDNA3.1-EIF5A2 was constructed as previously described [7]. MMP2 cDNA, purchased from GeneCopoeia Inc, was cloned into pcDNA3.1(+). Firefly luciferase reporter plasmids pGL3-MMP2-promoter-wild-type (WT) and pGL3-MMP2-ΔAP-1 contained wild-type and an AP-1 binding site (−1242 ~ −1239) deletion mutation of human MMP2 promoter, respectively. The wild type 3′-UTR segment of human MMP2 mRNA was cloned into the pMIR-REPORT luciferase reporter vector. The constructs was prepared according to standard clone protocol. The primers used for cloning are listed in Table 5.

**Table 5:** Primer sequences used for cloning

Name	Sequence
MMP2-p-F	5′-AGTAGATCT TTCTCCAGTGCCTCTTGCTG-3′
MMP2 -p-△AP1-F	5′-AGTAGATCTGAAGTCAGGCGTTCCCAAC-3′
MMP2-p-R	5′-AGTAAGCTT AGGTCCTGGCAATCCCTTTG-3′
MMP2-UTR-F	5′-TGAACTAGTCACTGCCTTCGATACACCG-3′
MMP2-UTR-R	5′-TCCAAGCTTCACGTGAAAAGTGCCTTGC-3′
MMP2-F	5′-TTACTCGAGATGGAGGCGCTAATGGCC-3′
MMP2-R	5′-TATACGCGTTCAGCAGCCTAGCCAGTC-3′

### Lentivirus production and transduction

Virus particles were harvested 48 h after shRNA specific for human EIF5A2 was transfected with the packaging plasmid pRSV/pREV, pCMV/pVSVG and pMDLG/pRRE into 293FTcells using Lipofectamine 2000 (Invitrogen). PLC8024 and Huh7 cells were infected with recombinant lentivirus-transducing units supplemented with 8 mg/ml Polybrene (Sigma, St Louis, Missouri, USA).

### Cell transfections

pcDNA3.1-EIF5A2 or the control plasmid pcDNA3.1(+) or short interfering RNAs (25nM) were transfected into HCC cells in 6-well plates using Lipofectamine 2000 (Invitrogen, Carlsbad, CA, USA) according to the manufacturer's instructions. The efficiency of transfection was measured by western blotting or gelatin zymography 48 h post-transfection.

### Cell invasion assay

The transwell cell invasion assay was performed in BD BioCoat Matrigel Invasion Chambers (Becton Dickinson Labware, Franklin Lakes, NJ) with 8μm pores according to the manufacturer's instructions. Briefly, 36 hours after transfection with RNA duplexes, 2×10^5^ tumor cells diluted in 100μl serum-free medium were added to the upper compartment of the chamber, while the lower compartment was filled with 500μl of DMEM containing 10% FBS. After 24 h, the non-invading cells were gently removed with a cotton swab. Invasive cells located on the lower side of the chamber were stained with crystal violet, air dried and photographed. Experiments were performed 3 times.

### Preparation of tumor cell-conditioned medium (TCM)

Liver tumor cells were first incubated in serum-free medium for 24h. Then, TCM was collected and centrifuged to remove cell debris. TCM was then stored in aliquots at -80ºC until used. Each corresponding well was subsequently trypsinized and the number of live cells was counted to allow appropriate correction of TCM loading. For the MMP-2 blocking assay, TCM was preincubated with an MMP-2-neutralizing antibody (ab80737, Abcam) or an isotype-matched control immunoglobulin G (IgG) (Boster, Wuhan, China) for 1 hour at 37ºC, before being applied to the HUVEC coculture.

### Capillary tube formation assay

HUVECs were cultured in the absence or presence of 75% TCM for 10 hours at 37ºC in a 96-well plate coated with Matrigel (R&D Systems, Minneapolis, MN). The formation of capillary-like structures was captured under a light microscope. The formed tubes, which represented the degree of *in vitro* angiogenesis, were scanned and quantitated in five low-power fields (100x).

### Nude mice xenograft studies

All experimental procedures involving animals were performed in accordance with the Guide for the Care and Use of Laboratory Animals (NIH publications 86-23, revised 1985), and according to the institutional ethical guidelines for animal experiments. For the subcutaneous xenograft model, PLC8024 cells (6×10^6^) that were transducted with NC or shRNA specific for EIF5A2 were suspended in 200μl 1xPBS and then injected subcutaneously into the right side the posterior flank of BALB/c athymic nude mice at 5 weeks of age. Twelve nude mice were injected and tumor growth was examined over the course of 35 days. Treatment with 5-FU in nude mice were performed as previously described [40]. In some experiments, local tumor blood flow was measured with a Laser Doppler imager (Perimed AB, Box564, SE-17526, Stockholm, Sweden) on anesthetized mice as previously described [41].

### Dual-luciferase assay

Liver cells grown in a 24-well plate were cotransfected with 400ng of either pGL3-MMP2-promoter-WT or pGL3-MMP2-ΔAP-1 or pMIR-MMP2-3′UTR and 10ng of pRL-TK (Promega, Madison, WI) and used for luciferase assays 48 h after transfection according to standard protocol. Each experiment was performed independently in triplicate.

### Gelatin zymography assay

Liver cells were incubated in serum-free DMEM for 24h. The TCM was collected, concentrated (Microcon 10K; Millipore Co., Bedford, MA), and mixed with 2X non-reducing sample buffer and directly applied to a 10% acrylamide gel containing 1 mg/ml gelatin. After electrophoresis, the gel was soaked in 2.5% Triton X-100 to remove SDS and then incubated at 37°C overnight in the development buffer, followed by staining with Coomassie Brilliant Blue R-250.

### Immunofluorescence (IF) staining

Tumor tissue sections were deparaffinized and rehydrated through graded alcohol, immersed in 3% hydrogen peroxide to block endogenous peroxidase activity, and subjected to antigen retrieval by microwaving and boiled in EDTA buffer (pH = 8). Subsequently, the slides were incubated with anti-mouse/human CD34 and collagen IV overnight at 4°C. After washing, slides underwent incubation with fluorescence-conjugated secondary antibodies. Finally, slides were washed and mounted with DAPI (Vector Laboratories, Burlingame, CA) before imaging using a fluorescence microscope (Leica, Deerfield, IL).

### Tumor hypoxia

Hypoxia in tumors was detected by the formation of pimonidazole adducts as previously described [42, 43].

### Tumor perfusion assay

Tumor vessel perfusion assays were performed as previously described [44]. Briefly, tumor-bearing mice were injected via the tail vein with Texas Red-conjugated Dextran at 100mg/kg (Sigma-Aldrich, Shanghai, China). After 3h, mice were injected with FITC-Lectin (Vector Laboratories, Burlingame, U.S.A.) at 10mg/kg. After ten minutes, mice were perfused with saline and tumors were harvested for cryosection and examined by fluorescence microscopy.

### Transmission electron microscope (TEM)

Excised tumor tissues were fixed immediately in 2% paraformaldehyde and 2.5% gluteraldehyde, after which tissues were cut into small pieces (0.5-1.0mm^3^) and fixed overnight at 4°C, then postfixed in 1% osmium tetroxide, dehydrated and embedded according to standard protocol. Ultra-thin sections were cut and stained with lead citrate and uranyl acetate and examined with a transmission electron microscope (Tecnai G2 Spirit Twin, FEI, U.S.A.).

### Immunohistochemistry (IHC) and selection of cut-point score

TMA slides were incubated respectively with anti-EIF5A2 (kindly provide by Dr Geng-Xi Hu, 1:100 dilution), anti-MMP2 and anti-CD34 (Santa Cruz, CA, 1:100 dilution). Immunostaining was performed using the Envision System with diaminobenzidine (Dako, Glostrup, Denmark). A negative control was obtained by replacing the primary antibody with a normal IgG. Known immunostaining positive cases were used as positive controls.

The immunoreactivity for EIF5A2 and MMP2 were scored in a semiquantitative method. In brief, each TMA spot was assigned an intensity score from 0–3 (I0, I1–3). Then, the proportion of tumor cells of that intensity was divided by the total number of tumor cells and recorded in 5% increments from 0 to 100 (P0, P1–3). The final H score (range 0–300) was determined by adding the sum of the scores obtained for each intensity and the proportion of the area stained (H score = [I1×P1]+[I2×P2]+[I3×P3]). The microvessel density (MVD) in tumor tissues was evaluated by IHC staining for CD34. Any discrete cluster or single cell stained for CD34 was counted as one microvessel. The scores were assessed by two independent pathologists (Cai MY and Xie D) who were blinded to the clinicopathological data.

X-tile plots were used for assessment of EIF5A2, MMP2 and CD34 expression and optimization of cut-point based on outcome. The X-tile plots allowed determination of an optimal cutoff value while correcting for the use of minimum *P* statistics by Miller-Siegmund *P*-value correction [45, 46].

### Statistical analysis

For survival analysis, the optimal cut-point for EIF5A2 expression was obtained using X-tile software version 3.6.1 (Yale University School of Medicine, New Haven, CT), as described previously [45]. The correlation between EIF5A2 expression and clinicopathological features of HCC patients was analyzed using the χ2 test or Fisher's exact test. For univariate survival analysis, survival curves were obtained using the Kaplan-Meier method. The Cox proportional hazards regression model was performed for multivariate survival analysis. The independent Student's t test was performed to analyze the statistical significance between two preselected groups. P values of less than 0.05 were considered statistically significant.

## SUPPLEMENTARY FIGURES


