# Supplementary material for: Ablation of EIF5A2 induces tumor vasculature remodeling and improves tumor response to chemotherapy via regulation of matrix metalloproteinase 2 expression
Source: Oncotarget. 2014 Jul 23;5(16):6716–33. doi: 10.18632/oncotarget.2236 (PMC4196158; doi:10.18632/oncotarget.2236)
Supplement: Supplementary file 1 [file oncotarget-05-6716-s001.pdf]

SUPPLEMENTARY FIGURES

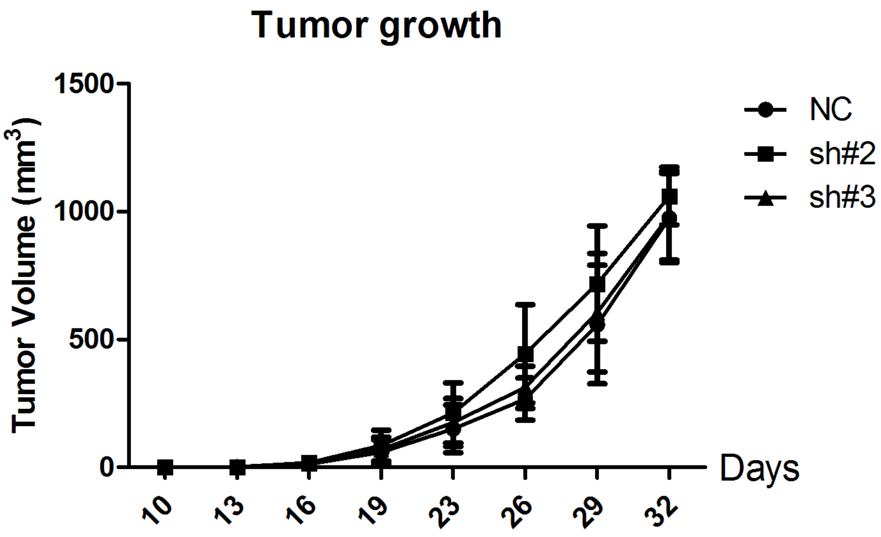

Supplementary Figure S1: Subcutaneous xenograft tumor growth curve of PLC8024 cells transfected with NC or shRNA-EIF5A2. Tumor volume (V) was monitored over a course of 35 days, by measuring the minor tumor axis (d) and the major tumor axis (D) of the tumor with calipers and calculated using the formula: $V = \pi x[d^2 xD]/6$.

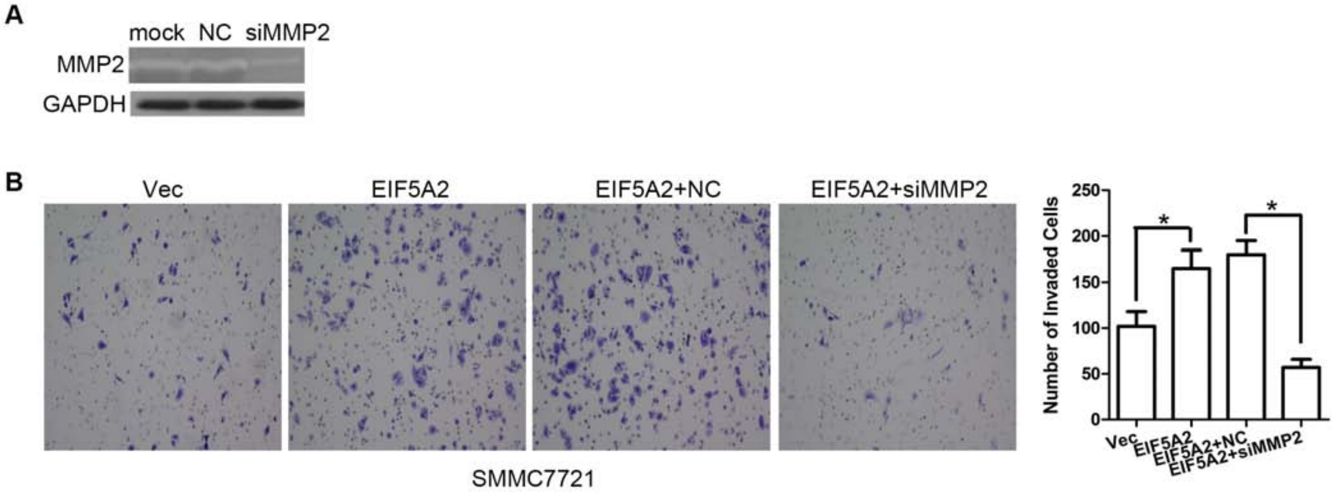

Supplementary Figure S2: Over-expression of EIF5A2 promotes HCC cell invasion. (A) MMP-2 activity in TCM examined by gelatin zymography. TCM was collected from cells transfected with MOCK, NC or si-RNA targeting MMP-2. **(B)** SMMC7721 cells transfected with indicated plasmid or si-RNA were applied to a transwell chamber coated with Matrigel and then incubated for 24h. Original magnification, 100x. *, $P<0.05$.

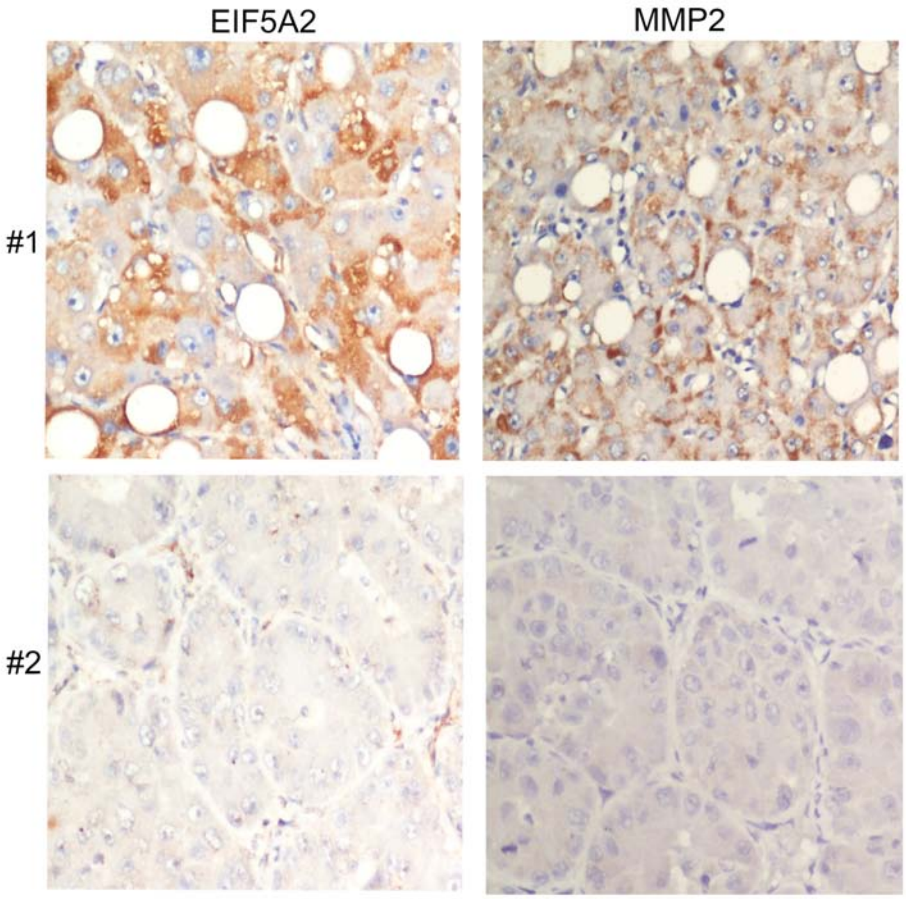

Supplementary Figure S3: Representative images of human HCC tissue sections stained for EIF5A2 and MMP-2 by IHC (100x). Case #1: high expression of EIF5A2 and MMP-2; Case #2: low expression of EIF5A2 and MMP-2.

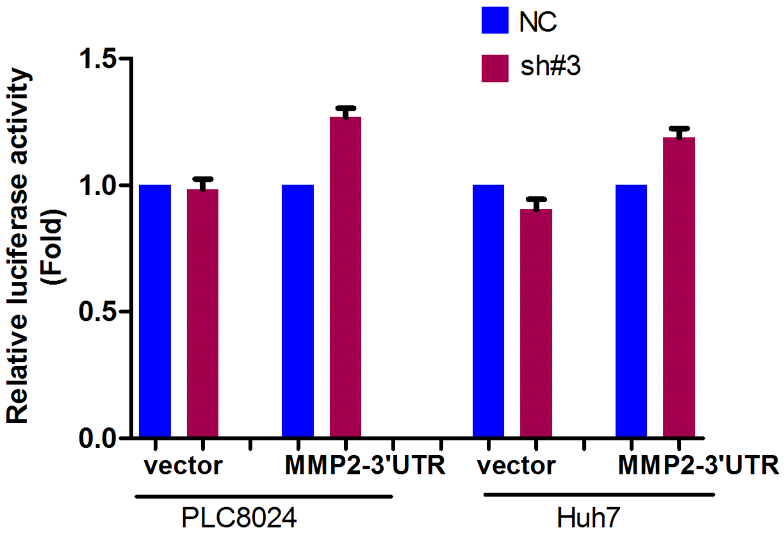

Supplementary Figure S4: Luciferase activity of the MMP-2 3’UTR reporter vector in indicated cells. MMP-2 3’UTR reporter vector and pRL-TK were cotransfected into liver cells then used in a luciferase assay 48 h after transfection. The experiments were performed independently in triplicate.
